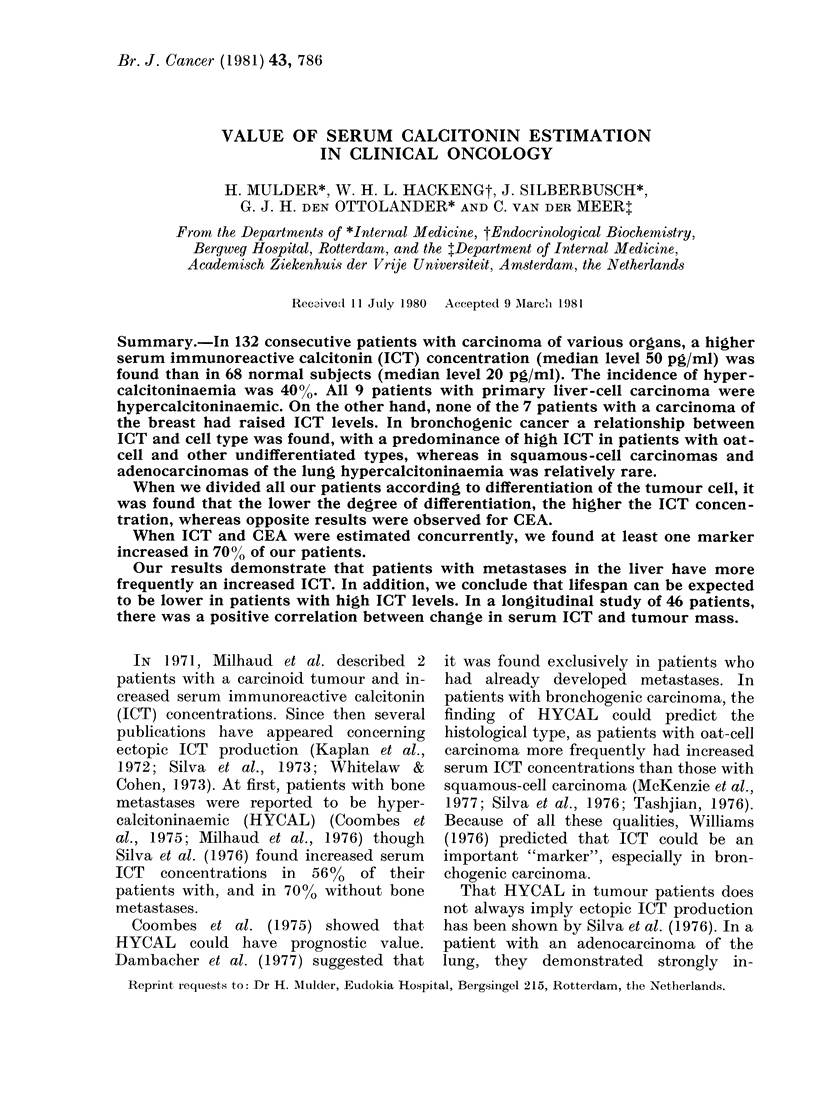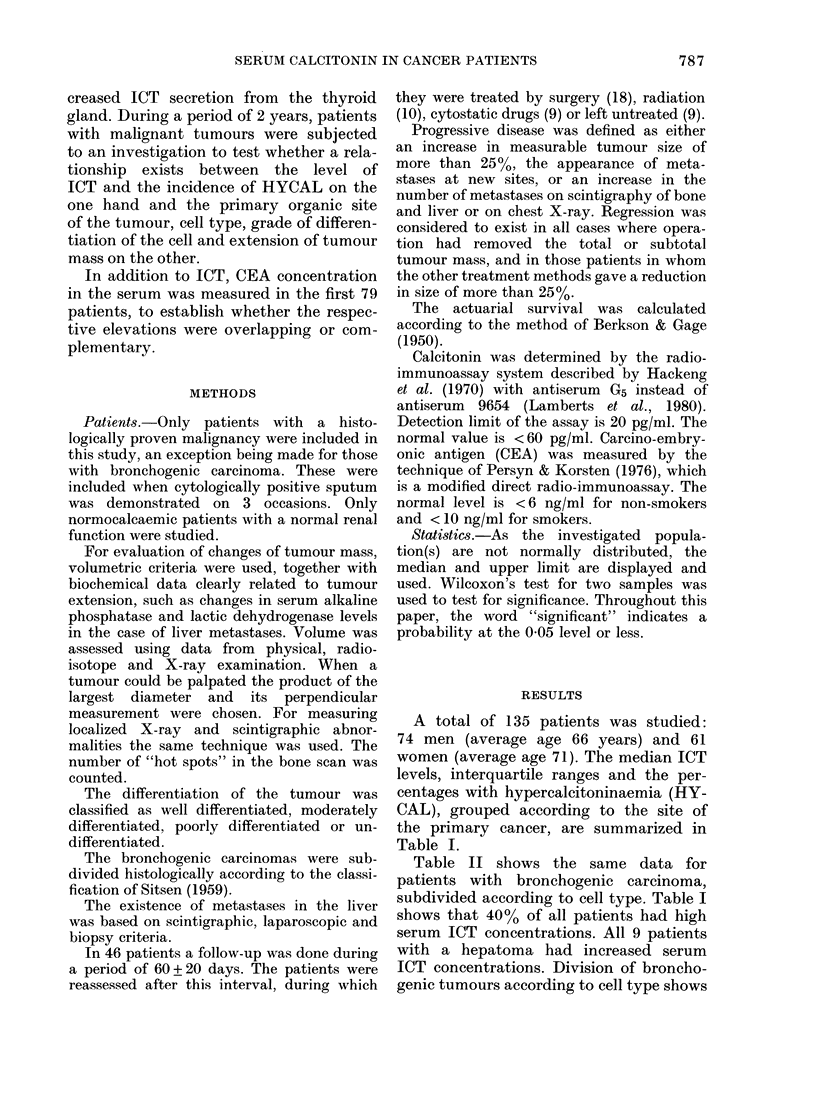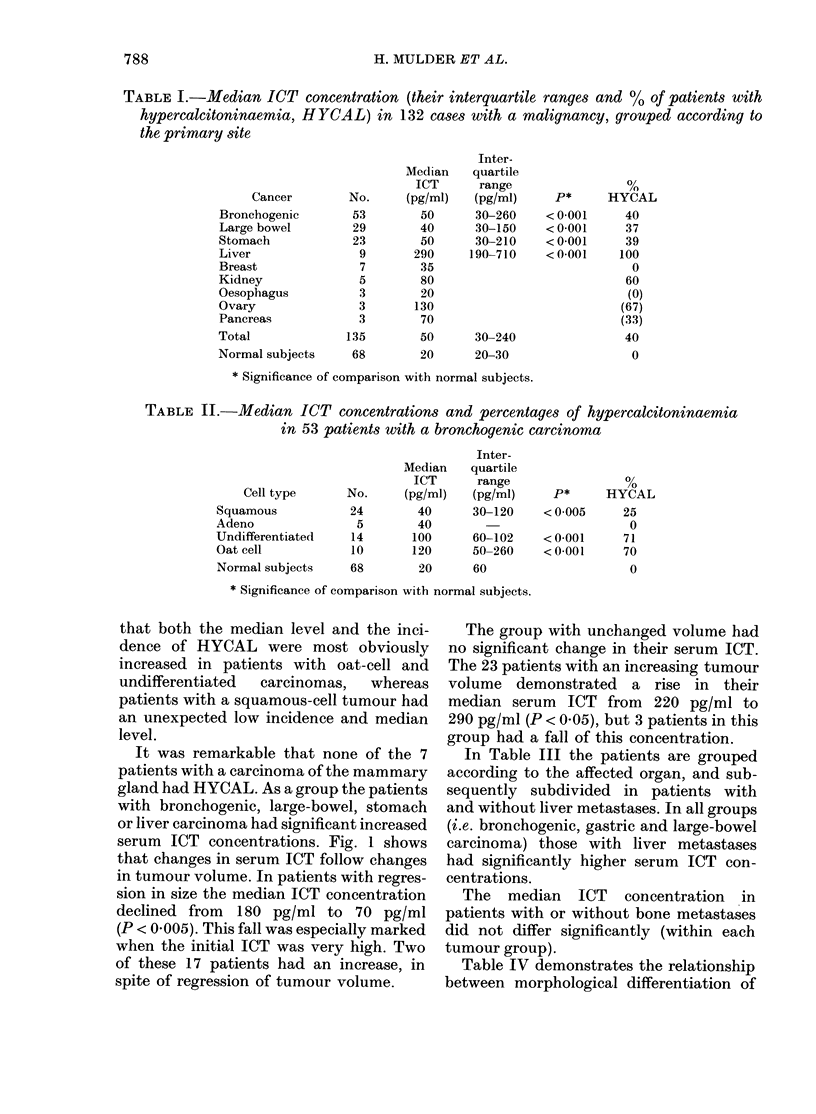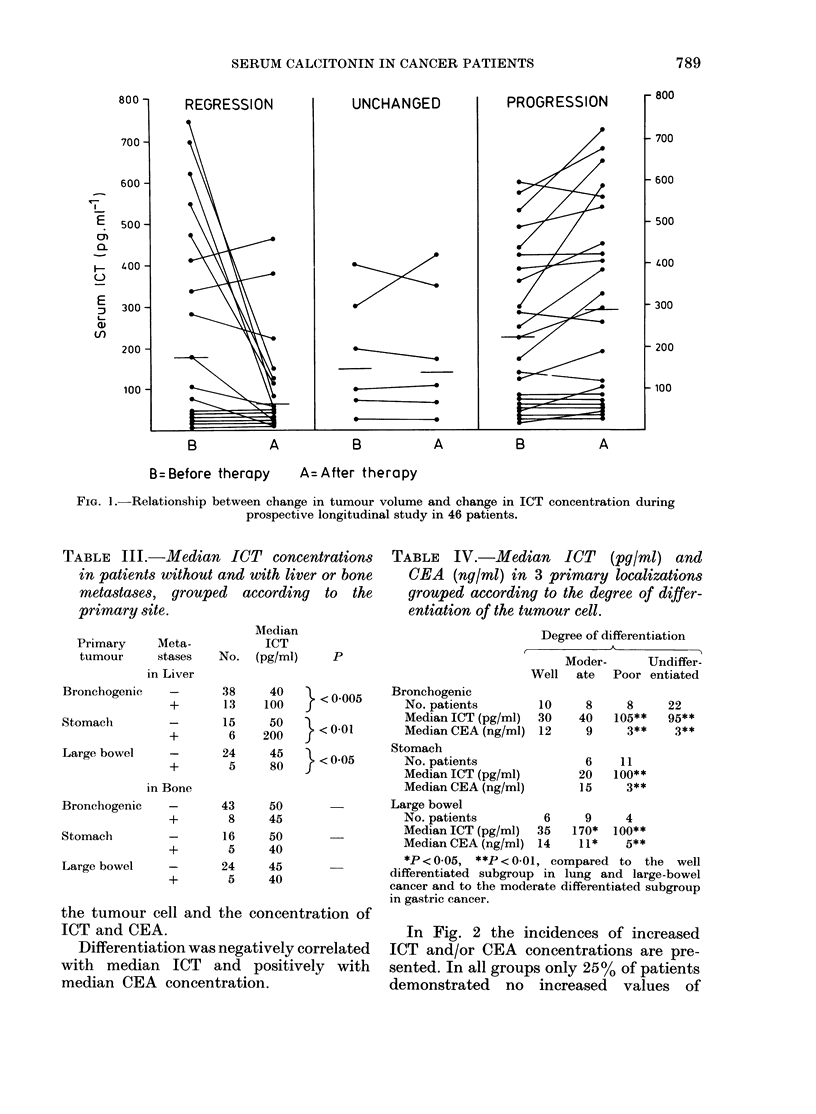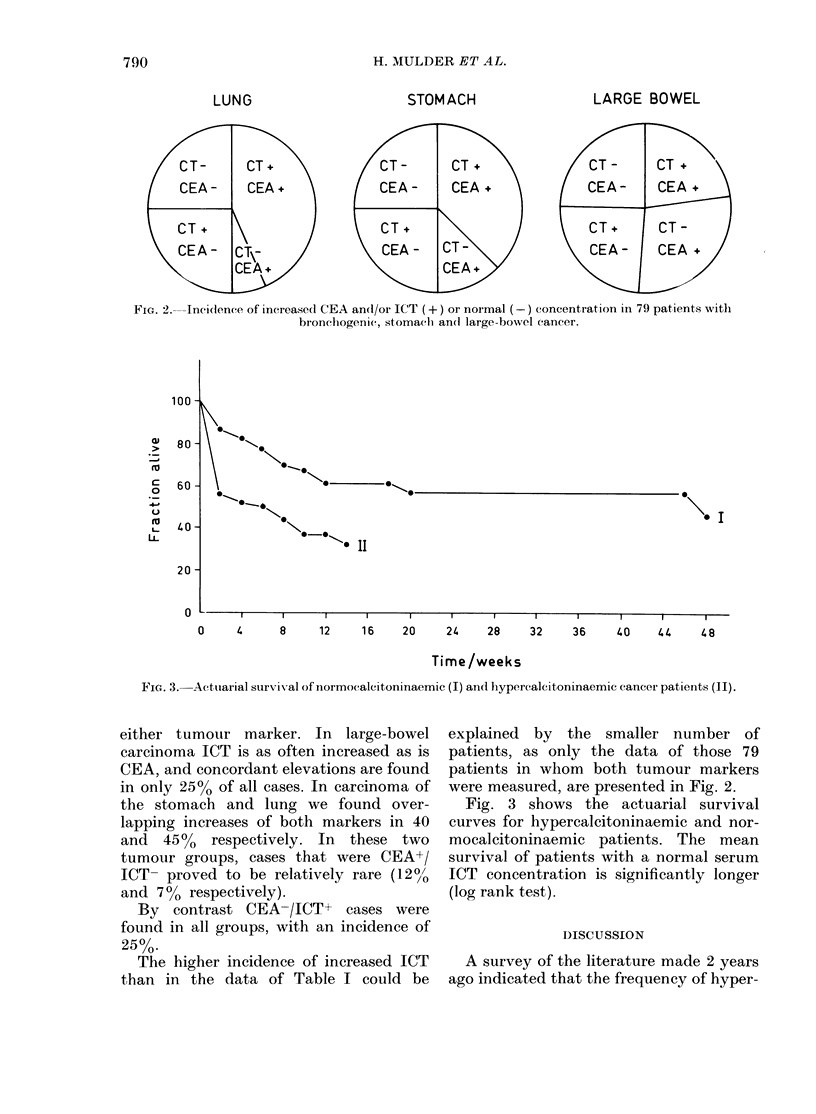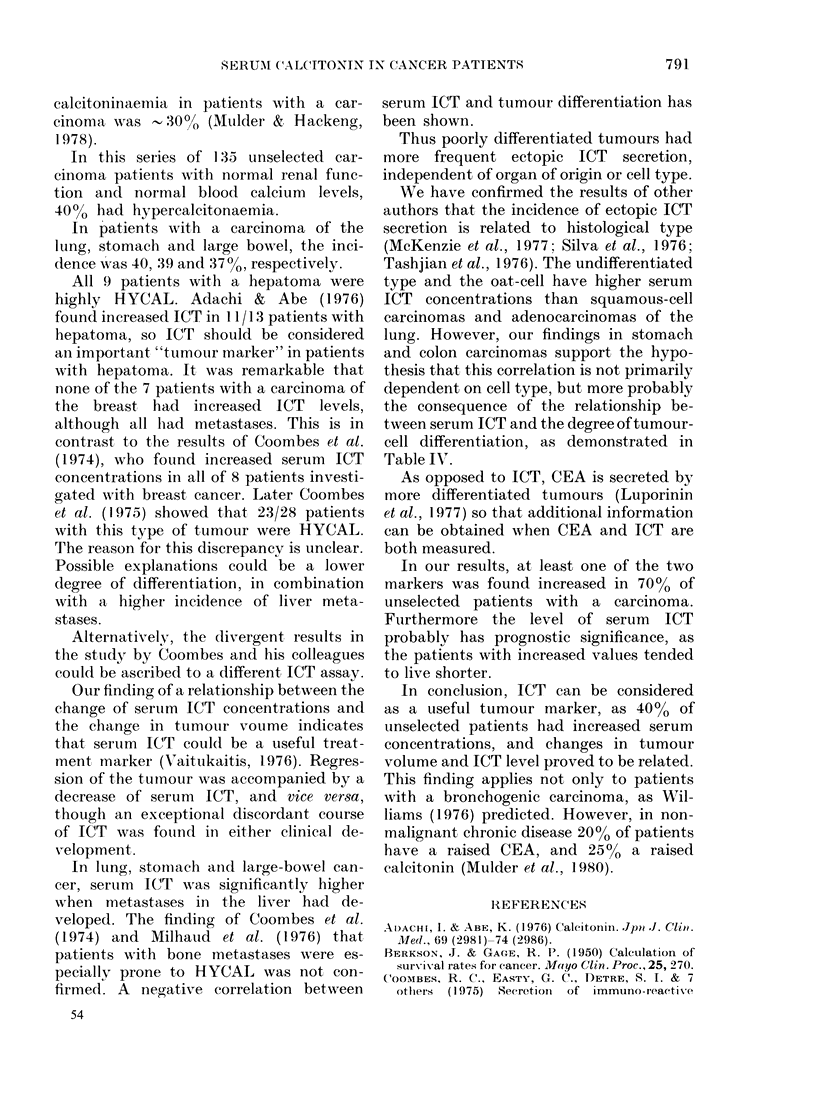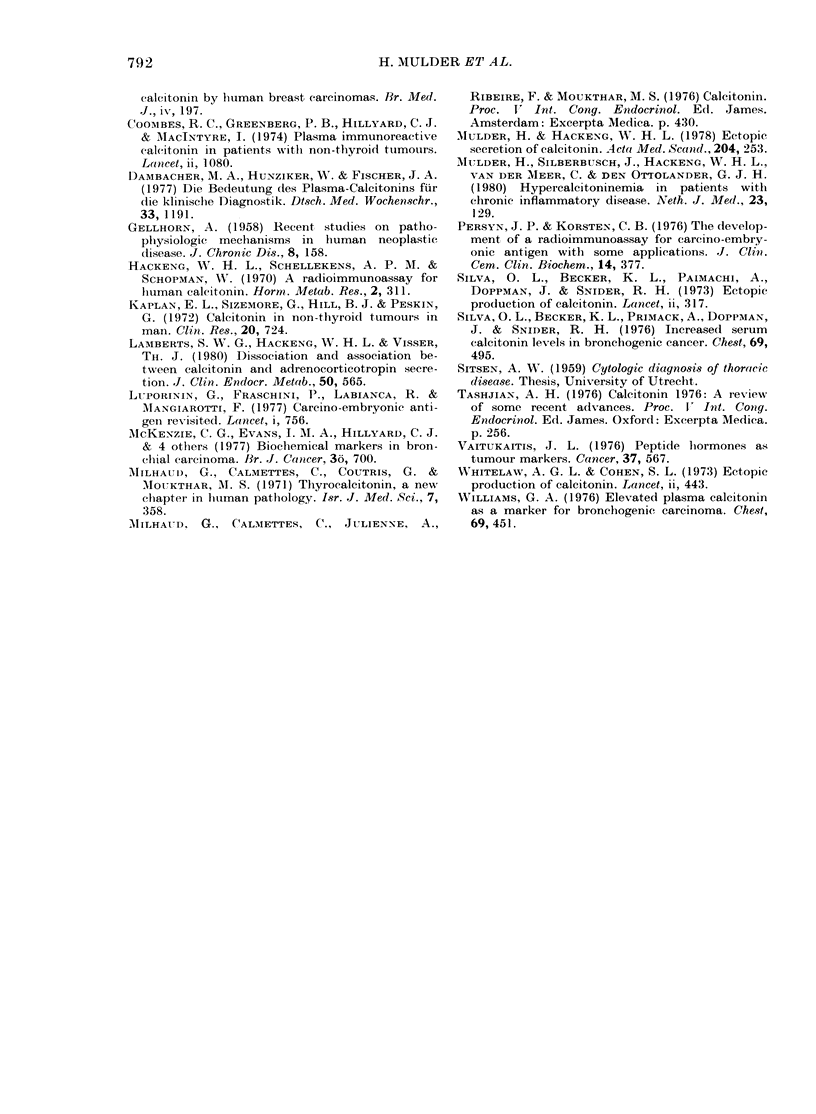# Value of serum calcitonin estimation in clinical oncology.

**DOI:** 10.1038/bjc.1981.116

**Published:** 1981-06

**Authors:** H. Mulder, W. H. Hackeng, J. Silberbusch, G. J. den Ottolander, C. van der Meer

## Abstract

In 132 consecutive patients with carcinoma of various organs, a higher serum immunoreactive calcitonin (ICT) concentration (median level 50 pg/ml) was found than in 68 normal subjects (median level 20 pg/ml). The incidence of hypercalcitoninaemia was 40%. All 9 patients with primary liver-cell carcinoma were hypercalcitoninaemic. On the other hand, none of the 7 patients with a carcinoma of the breast had raised ICT levels. In bronchogenic cancer a relationship between ICT and cell type was found, with a predominance of high ICT in patients with oatcell and other undifferentiated types, whereas in squamous-cell carcinomas and adenocarcinomas of the lung hypercalcitoninaemia was relatively rare. When we divided all our patients according to differentiation of the tumour cell, it was found that the lower the degree of differentiation, the higher the ICT concentration, whereas opposite results were observed for CEA. When ICT and CEA were estimated concurrently, we found at least one marker increased in 70% of our patients. Our results demonstrate that patients with metastases in the liver have more frequently and increased ICT. In addition, we conclude that lifespan can be expected to be lower in patients with high ICT levels. In a longitudinal study of 46 patients, there was a positive correlation between change in serum ICT and tumour mass.


*Br. J. Cancer* (1981) **43**, 786

### VALUE OF SERUM CALCITONIN ESTIMATION IN CLINICAL ONCOLOGY

H. MULDER*, W. H. L. HACKENG†, J. SILBERBUSCH*,
G. J. H. DEN OTTOLANDER* AND C. VAN DER MEER‡

*From the Departments of *Internal Medicine, †Endocrinological Biochemistry, Bergweg Hospital, Rotterdam, and the ‡Department of Internal Medicine, Academisch Ziekenhuis der Vrije Universiteit, Amsterdam, the Netherlands*

Received 11 July 1980 Accepted 9 March 1981

**Summary.—In 132 consecutive patients with carcinoma of various organs, a higher serum immunoreactive calcitonin (ICT) concentration (median level 50 pg/ml) was found than in 68 normal subjects (median level 20 pg/ml). The incidence of hypercalcitoninaemia was 40%. All 9 patients with primary liver-cell carcinoma were hypercalcitoninaemic. On the other hand, none of the 7 patients with a carcinoma of the breast had raised ICT levels. In bronchogenic cancer a relationship between ICT and cell type was found, with a predominance of high ICT in patients with oat-cell and other undifferentiated types, whereas in squamous-cell carcinomas and adenocarcinomas of the lung hypercalcitoninaemia was relatively rare.**

**When we divided all our patients according to differentiation of the tumour cell, it was found that the lower the degree of differentiation, the higher the ICT concentration, whereas opposite results were observed for CEA.**

**When ICT and CEA were estimated concurrently, we found at least one marker increased in 70% of our patients.**

**Our results demonstrate that patients with metastases in the liver have more frequently an increased ICT. In addition, we conclude that lifespan can be expected to be lower in patients with high ICT levels. In a longitudinal study of 46 patients, there was a positive correlation between change in serum ICT and tumour mass.**

IN 1971, Milhaud *et al.* described 2 patients with a carcinoid tumour and increased serum immunoreactive calcitonin (ICT) concentrations. Since then several publications have appeared concerning ectopic ICT production (Kaplan *et al.*, 1972; Silva *et al.*, 1973; Whitelaw & Cohen, 1973). At first, patients with bone metastases were reported to be hypercalcitoninaemic (HYCAL) (Coombes *et al.*, 1975; Milhaud *et al.*, 1976) though Silva *et al.* (1976) found increased serum ICT concentrations in 56% of their patients with, and in 70% without bone metastases.

Coombes *et al.* (1975) showed that HYCAL could have prognostic value. Dambacher *et al.* (1977) suggested that it was found exclusively in patients who had already developed metastases. In patients with bronchogenic carcinoma, the finding of HYCAL could predict the histological type, as patients with oat-cell carcinoma more frequently had increased serum ICT concentrations than those with squamous-cell carcinoma (McKenzie *et al.*, 1977; Silva *et al.*, 1976; Tashjian, 1976). Because of all these qualities, Williams (1976) predicted that ICT could be an important "marker", especially in bronchogenic carcinoma.

That HYCAL in tumour patients does not always imply ectopic ICT production has been shown by Silva *et al.* (1976). In a patient with an adenocarcinoma of the lung, they demonstrated strongly in-

Reprint requests to: Dr H. Mulder, Eudokia Hospital, Bergsingel 215, Rotterdam, the Netherlands.

creased ICT secretion from the thyroid gland. During a period of 2 years, patients with malignant tumours were subjected to an investigation to test whether a relationship exists between the level of ICT and the incidence of HYCAL on the one hand and the primary organic site of the tumour, cell type, grade of differentiation of the cell and extension of tumour mass on the other.

In addition to ICT, CEA concentration in the serum was measured in the first 79 patients, to establish whether the respective elevations were overlapping or complementary.

### METHODS

*Patients.*—Only patients with a histologically proven malignancy were included in this study, an exception being made for those with bronchogenic carcinoma. These were included when cytologically positive sputum was demonstrated on 3 occasions. Only normocalcaemic patients with a normal renal function were studied.

For evaluation of changes of tumour mass, volumetric criteria were used, together with biochemical data clearly related to tumour extension, such as changes in serum alkaline phosphatase and lactic dehydrogenase levels in the case of liver metastases. Volume was assessed using data from physical, radioisotope and X-ray examination. When a tumour could be palpated the product of the largest diameter and its perpendicular measurement were chosen. For measuring localized X-ray and scintigraphic abnormalities the same technique was used. The number of "hot spots" in the bone scan was counted.

The differentiation of the tumour was classified as well differentiated, moderately differentiated, poorly differentiated or undifferentiated.

The bronchogenic carcinomas were subdivided histologically according to the classification of Sitsen (1959).

The existence of metastases in the liver was based on scintigraphic, laparoscopic and biopsy criteria.

In 46 patients a follow-up was done during a period of $60 \pm 20$ days. The patients were reassessed after this interval, during which they were treated by surgery (18), radiation (10), cytostatic drugs (9) or left untreated (9).

Progressive disease was defined as either an increase in measurable tumour size of more than 25%, the appearance of metastases at new sites, or an increase in the number of metastases on scintigraphy of bone and liver or on chest X-ray. Regression was considered to exist in all cases where operation had removed the total or subtotal tumour mass, and in those patients in whom the other treatment methods gave a reduction in size of more than 25%.

The actuarial survival was calculated according to the method of Berkson & Gage (1950).

Calcitonin was determined by the radioimmunoassay system described by Hackeng *et al.* (1970) with antiserum $G_5$ instead of antiserum 9654 (Lamberts *et al.*, 1980). Detection limit of the assay is 20 pg/ml. The normal value is $<60$ pg/ml. Carcino-embryonic antigen (CEA) was measured by the technique of Persyn & Korsten (1976), which is a modified direct radio-immunoassay. The normal level is $<6$ ng/ml for non-smokers and $<10$ ng/ml for smokers.

*Statistics.*—As the investigated population(s) are not normally distributed, the median and upper limit are displayed and used. Wilcoxon's test for two samples was used to test for significance. Throughout this paper, the word "significant" indicates a probability at the 0·05 level or less.

### RESULTS

A total of 135 patients was studied: 74 men (average age 66 years) and 61 women (average age 71). The median ICT levels, interquartile ranges and the percentages with hypercalcitoninaemia (HYCAL), grouped according to the site of the primary cancer, are summarized in Table I.

Table II shows the same data for patients with bronchogenic carcinoma, subdivided according to cell type. Table I shows that 40% of all patients had high serum ICT concentrations. All 9 patients with a hepatoma had increased serum ICT concentrations. Division of bronchogenic tumours according to cell type shows

TABLE I.—*Median ICT concentration (their interquartile ranges and % of patients with hypercalcitoninaemia, HYCAL) in 132 cases with a malignancy, grouped according to the primary site*

| Cancer | No. | Median ICT (pg/ml) | Inter-quartile range (pg/ml) | P* | % HYCAL |
|---|---|---|---|---|---|
| Bronchogenic | 53 | 50 | 30–260 | < 0·001 | 40 |
| Large bowel | 29 | 40 | 30–150 | < 0·001 | 37 |
| Stomach | 23 | 50 | 30–210 | < 0·001 | 39 |
| Liver | 9 | 290 | 190–710 | < 0·001 | 100 |
| Breast | 7 | 35 | | | 0 |
| Kidney | 5 | 80 | | | 60 |
| Oesophagus | 3 | 20 | | | (0) |
| Ovary | 3 | 130 | | | (67) |
| Pancreas | 3 | 70 | | | (33) |
| Total | 135 | 50 | 30–240 | | 40 |
| Normal subjects | 68 | 20 | 20–30 | | 0 |

* Significance of comparison with normal subjects.

TABLE II.—*Median ICT concentrations and percentages of hypercalcitoninaemia in 53 patients with a bronchogenic carcinoma*

| Cell type | No. | Median ICT (pg/ml) | Inter-quartile range (pg/ml) | P* | % HYCAL |
|---|---|---|---|---|---|
| Squamous | 24 | 40 | 30–120 | < 0·005 | 25 |
| Adeno | 5 | 40 | — | | 0 |
| Undifferentiated | 14 | 100 | 60–102 | < 0·001 | 71 |
| Oat cell | 10 | 120 | 50–260 | < 0·001 | 70 |
| Normal subjects | 68 | 20 | 60 | | 0 |

* Significance of comparison with normal subjects.

that both the median level and the incidence of HYCAL were most obviously increased in patients with oat-cell and undifferentiated carcinomas, whereas patients with a squamous-cell tumour had an unexpected low incidence and median level.

It was remarkable that none of the 7 patients with a carcinoma of the mammary gland had HYCAL. As a group the patients with bronchogenic, large-bowel, stomach or liver carcinoma had significant increased serum ICT concentrations. Fig. 1 shows that changes in serum ICT follow changes in tumour volume. In patients with regression in size the median ICT concentration declined from 180 pg/ml to 70 pg/ml ($P < 0{\cdot}005$). This fall was especially marked when the initial ICT was very high. Two of these 17 patients had an increase, in spite of regression of tumour volume.

The group with unchanged volume had no significant change in their serum ICT. The 23 patients with an increasing tumour volume demonstrated a rise in their median serum ICT from 220 pg/ml to 290 pg/ml ($P < 0{\cdot}05$), but 3 patients in this group had a fall of this concentration.

In Table III the patients are grouped according to the affected organ, and subsequently subdivided in patients with and without liver metastases. In all groups (*i.e.* bronchogenic, gastric and large-bowel carcinoma) those with liver metastases had significantly higher serum ICT concentrations.

The median ICT concentration in patients with or without bone metastases did not differ significantly (within each tumour group).

Table IV demonstrates the relationship between morphological differentiation of

FIG. 1.—Relationship between change in tumour volume and change in ICT concentration during prospective longitudinal study in 46 patients.

TABLE III.—*Median ICT concentrations in patients without and with liver or bone metastases, grouped according to the primary site.*

| Primary tumour | Metastases | No. | Median ICT (pg/ml) | P |
|---|---|---|---|---|
| | in Liver | | | |
| Bronchogenic | − | 38 | 40 | < 0·005 |
| | + | 13 | 100 | |
| Stomach | − | 15 | 50 | < 0·01 |
| | + | 6 | 200 | |
| Large bowel | − | 24 | 45 | < 0·05 |
| | + | 5 | 80 | |
| | in Bone | | | |
| Bronchogenic | − | 43 | 50 | — |
| | + | 8 | 45 | |
| Stomach | − | 16 | 50 | — |
| | + | 5 | 40 | |
| Large bowel | − | 24 | 45 | — |
| | + | 5 | 40 | |

the tumour cell and the concentration of ICT and CEA.

Differentiation was negatively correlated with median ICT and positively with median CEA concentration.

TABLE IV.—*Median ICT (pg/ml) and CEA (ng/ml) in 3 primary localizations grouped according to the degree of differentiation of the tumour cell.*

| | Degree of differentiation: Well | Moderate | Poor | Undifferentiated |
|---|---|---|---|---|
| Bronchogenic | | | | |
| No. patients | 10 | 8 | 8 | 22 |
| Median ICT (pg/ml) | 30 | 40 | 105** | 95** |
| Median CEA (ng/ml) | 12 | 9 | 3** | 3** |
| Stomach | | | | |
| No. patients | | 6 | 11 | |
| Median ICT (pg/ml) | | 20 | 100** | |
| Median CEA (ng/ml) | | 15 | 3** | |
| Large bowel | | | | |
| No. patients | 6 | 9 | 4 | |
| Median ICT (pg/ml) | 35 | 170* | 100** | |
| Median CEA (ng/ml) | 14 | 11* | 5** | |

$^*P < 0{\cdot}05$, $^{**}P < 0{\cdot}01$, compared to the well differentiated subgroup in lung and large-bowel cancer and to the moderate differentiated subgroup in gastric cancer.

In Fig. 2 the incidences of increased ICT and/or CEA concentrations are presented. In all groups only 25% of patients demonstrated no increased values of

FIG. 2.—Incidence of increased CEA and/or ICT (+) or normal (−) concentration in 79 patients with bronchogenic, stomach and large-bowel cancer.

FIG. 3.—Actuarial survival of normocalcitoninaemic (I) and hypercalcitoninaemic cancer patients (II).

either tumour marker. In large-bowel carcinoma ICT is as often increased as is CEA, and concordant elevations are found in only 25% of all cases. In carcinoma of the stomach and lung we found overlapping increases of both markers in 40 and 45% respectively. In these two tumour groups, cases that were $CEA^+$/$ICT^-$ proved to be relatively rare (12% and 7% respectively).

By contrast $CEA^-$/$ICT^+$ cases were found in all groups, with an incidence of 25%.

The higher incidence of increased ICT than in the data of Table I could be explained by the smaller number of patients, as only the data of those 79 patients in whom both tumour markers were measured, are presented in Fig. 2.

Fig. 3 shows the actuarial survival curves for hypercalcitoninaemic and normocalcitoninaemic patients. The mean survival of patients with a normal serum ICT concentration is significantly longer (log rank test).

### DISCUSSION

A survey of the literature made 2 years ago indicated that the frequency of hypercalcitoninaemia in patients with a carcinoma was ~30% (Mulder & Hackeng, 1978).

In this series of 135 unselected carcinoma patients with normal renal function and normal blood calcium levels, 40% had hypercalcitonaemia.

In patients with a carcinoma of the lung, stomach and large bowel, the incidence was 40, 39 and 37%, respectively.

All 9 patients with a hepatoma were highly HYCAL. Adachi & Abe (1976) found increased ICT in 11/13 patients with hepatoma, so ICT should be considered an important "tumour marker" in patients with hepatoma. It was remarkable that none of the 7 patients with a carcinoma of the breast had increased ICT levels, although all had metastases. This is in contrast to the results of Coombes *et al.* (1974), who found increased serum ICT concentrations in all of 8 patients investigated with breast cancer. Later Coombes *et al.* (1975) showed that 23/28 patients with this type of tumour were HYCAL. The reason for this discrepancy is unclear. Possible explanations could be a lower degree of differentiation, in combination with a higher incidence of liver metastases.

Alternatively, the divergent results in the study by Coombes and his colleagues could be ascribed to a different ICT assay.

Our finding of a relationship between the change of serum ICT concentrations and the change in tumour voume indicates that serum ICT could be a useful treatment marker (Vaitukaitis, 1976). Regression of the tumour was accompanied by a decrease of serum ICT, and *vice versa*, though an exceptional discordant course of ICT was found in either clinical development.

In lung, stomach and large-bowel cancer, serum ICT was significantly higher when metastases in the liver had developed. The finding of Coombes *et al.* (1974) and Milhaud *et al.* (1976) that patients with bone metastases were especially prone to HYCAL was not confirmed. A negative correlation between serum ICT and tumour differentiation has been shown.

Thus poorly differentiated tumours had more frequent ectopic ICT secretion, independent of organ of origin or cell type.

We have confirmed the results of other authors that the incidence of ectopic ICT secretion is related to histological type (McKenzie *et al.*, 1977; Silva *et al.*, 1976; Tashjian *et al.*, 1976). The undifferentiated type and the oat-cell have higher serum ICT concentrations than squamous-cell carcinomas and adenocarcinomas of the lung. However, our findings in stomach and colon carcinomas support the hypothesis that this correlation is not primarily dependent on cell type, but more probably the consequence of the relationship between serum ICT and the degree of tumour-cell differentiation, as demonstrated in Table IV.

As opposed to ICT, CEA is secreted by more differentiated tumours (Luporinin *et al.*, 1977) so that additional information can be obtained when CEA and ICT are both measured.

In our results, at least one of the two markers was found increased in 70% of unselected patients with a carcinoma. Furthermore the level of serum ICT probably has prognostic significance, as the patients with increased values tended to live shorter.

In conclusion, ICT can be considered as a useful tumour marker, as 40% of unselected patients had increased serum concentrations, and changes in tumour volume and ICT level proved to be related. This finding applies not only to patients with a bronchogenic carcinoma, as Williams (1976) predicted. However, in non-malignant chronic disease 20% of patients have a raised CEA, and 25% a raised calcitonin (Mulder *et al.*, 1980).

### REFERENCES

ADACHI, I. & ABE, K. (1976) Calcitonin. *Jpn J. Clin. Med.*, 69 (2981)–74 (2986).

BERKSON, J. & GAGE, R. P. (1950) Calculation of survival rates for cancer. *Mayo Clin. Proc.*, **25**, 270.

COOMBES, R. C., EASTY, G. C., DETRE, S. I. & 7 others (1975) Secretion of immuno-reactive calcitonin by human breast carcinomas. *Br. Med. J.*, iv, 197.

COOMBES, R. C., GREENBERG, P. B., HILLYARD, C. J. & MACINTYRE, I. (1974) Plasma immunoreactive calcitonin in patients with non-thyroid tumours. *Lancet*, ii, 1080.

DAMBACHER, M. A., HUNZIKER, W. & FISCHER, J. A. (1977) Die Bedeutung des Plasma-Calcitonins für die klinische Diagnostik. *Dtsch. Med. Wochenschr.*, **33,** 1191.

GELLHORN, A. (1958) Recent studies on pathophysiologic mechanisms in human neoplastic disease. *J. Chronic Dis.*, **8,** 158.

HACKENG, W. H. L., SCHELLEKENS, A. P. M. & SCHOPMAN, W. (1970) A radioimmunoassay for human calcitonin. *Horm. Metab. Res.*, **2,** 311.

KAPLAN, E. L., SIZEMORE, G., HILL, B. J. & PESKIN, G. (1972) Calcitonin in non-thyroid tumours in man. *Clin. Res.*, **20,** 724.

LAMBERTS, S. W. G., HACKENG, W. H. L. & VISSER, TH. J. (1980) Dissociation and association between calcitonin and adrenocorticotropin secretion. *J. Clin. Endocr. Metab.*, **50,** 565.

LUPORININ, G., FRASCHINI, P., LABIANCA, R. & MANGIAROTTI, F. (1977) Carcino-embryonic antigen revisited. *Lancet*, i, 756.

MCKENZIE, C. G., EVANS, I. M. A., HILLYARD, C. J. & 4 others (1977) Biochemical markers in bronchial carcinoma. *Br. J. Cancer*, **3ö,** 700.

MILHAUD, G., CALMETTES, C., COUTRIS, G. & MOUKTHAR, M. S. (1971) Thyrocalcitonin, a new chapter in human pathology. *Isr. J. Med. Sci.*, **7,** 358.

MILHAUD, G., CALMETTES, C., JULIENNE, A., RIBEIRE, F. & MOUKTHAR, M. S. (1976) Calcitonin. *Proc. V Int. Cong. Endocrinol.* Ed. James. Amsterdam: Excerpta Medica. p. 430.

MULDER, H. & HACKENG, W. H. L. (1978) Ectopic secretion of calcitonin. *Acta Med. Scand.*, **204,** 253.

MULDER, H., SILBERBUSCH, J., HACKENG, W. H. L., VAN DER MEER, C. & DEN OTTOLANDER, G. J. H. (1980) Hypercalcitoninemia in patients with chronic inflammatory disease. *Neth. J. Med.*, **23,** 129.

PERSYN, J. P. & KORSTEN, C. B. (1976) The development of a radioimmunoassay for carcino-embryonic antigen with some applications. *J. Clin. Cem. Clin. Biochem.*, **14,** 377.

SILVA, O. L., BECKER, K. L., PAIMACHI, A., DOPPMAN, J. & SNIDER, R. H. (1973) Ectopic production of calcitonin. *Lancet*, ii, 317.

SILVA, O. L., BECKER, K. L., PRIMACK, A., DOPPMAN, J. & SNIDER, R. H. (1976) Increased serum calcitonin levels in bronchogenic cancer. *Chest*, **69,** 495.

SITSEN, A. W. (1959) *Cytologic diagnosis of thoracic disease.* Thesis, University of Utrecht.

TASHJIAN, A. H. (1976) Calcitonin 1976: A review of some recent advances. *Proc. V Int. Cong. Endocrinol.* Ed. James. Oxford: Excerpta Medica. p. 256.

VAITUKAITIS, J. L. (1976) Peptide hormones as tumour markers. *Cancer*, **37,** 567.

WHITELAW, A. G. L. & COHEN, S. L. (1973) Ectopic production of calcitonin. *Lancet*, ii, 443.

WILLIAMS, G. A. (1976) Elevated plasma calcitonin as a marker for bronchogenic carcinoma. *Chest*, **69,** 451.